# Crozier’s paradox revisited: maintenance of genetic recognition systems by disassortative mating

**DOI:** 10.1186/1471-2148-13-211

**Published:** 2013-09-27

**Authors:** Luke Holman, Jelle S van Zweden, Timothy A Linksvayer, Patrizia d’Ettorre

**Affiliations:** 1 Department of Biology, Centre for Social Evolution, University of Copenhagen, Universitetsparken 15, Copenhagen 2100, Denmark; 2 Present address: Division of Evolution, Ecology and Genetics, Research School of Biology, The Australian National University, Canberra, ACT 0200, Australia; 3 Present address: Laboratory of Socioecology & Social Evolution (LSSE), University of Leuven, Naamsestraat 59, Leuven 3000, Belgium; 4 Present address: Department of Biology, University of Pennsylvania, 415 S. University Avenue, Philadelphia 19104 PA, USA; 5 Present address: Laboratoire d’Ethologie Expérimentale et Comparée (LEEC), University of Paris 13, 99 av. J.B. Clément, Villetaneuse F-93430, France

**Keywords:** Altruism, Frequency-dependent selection, Green beard, Kin discrimination, Sexual selection

## Abstract

**Background:**

Organisms are predicted to behave more favourably towards relatives, and kin-biased cooperation has been found in all domains of life from bacteria to vertebrates. Cooperation based on genetic recognition cues is paradoxical because it disproportionately benefits individuals with common phenotypes, which should erode the required cue polymorphism. Theoretical models suggest that many recognition loci likely have some secondary function that is subject to diversifying selection, keeping them variable.

**Results:**

Here, we use individual-based simulations to investigate the hypothesis that the dual use of recognition cues to facilitate social behaviour and disassortative mating (e.g. for inbreeding avoidance) can maintain cue diversity over evolutionary time. Our model shows that when organisms mate disassortatively with respect to their recognition cues, cooperation and recognition locus diversity can persist at high values, especially when outcrossed matings produce more surviving offspring. Mating system affects cue diversity via at least four distinct mechanisms, and its effects interact with other parameters such as population structure. Also, the attrition of cue diversity is less rapid when cooperation does not require an exact cue match. Using a literature review, we show that there is abundant empirical evidence that heritable recognition cues are simultaneously used in social and sexual behaviour.

**Conclusions:**

Our models show that mate choice is one possible resolution of the paradox of genetic kin recognition, and the literature review suggests that genetic recognition cues simultaneously inform assortative cooperation and disassortative mating in a large range of taxa. However, direct evidence is scant and there is substantial scope for future work.

## Background

Cooperation is predicted to evolve more easily when social partners are genetically related [1-5]. In many species, individuals are able to assess the genetic similarity of conspecifics using heritable phenotypic cues, for example by comparing their own cues to those of their social partners (self-referent phenotype matching; [6]), or by directly identifying individuals with the same genotype as them at a particular locus ("green beard" recognition; [7]). Such cue-dependent cooperation is thought to be common in all domains of life, including plants [8], fungi [9], bacteria [10], vertebrates [11], insects [12], slime moulds [13] and sessile marine invertebrates [14].

Cooperation based on genetic cues is a conundrum because it requires polymorphic recognition loci, yet cooperation is predicted to erode this genetic variation (e.g. [15-17]). This problem, sometimes termed Crozier’s paradox, applies whenever individuals with common recognition cues receive greater average fitness returns from social interactions. For example, individuals with common cues might be aggressively rejected less often [15,18,19], or might receive altruism from a greater proportion of the population [17,20]. Disproportionate fitness benefits for individuals with common recognition alleles should produce positive frequency-dependent selection at recognition loci, depleting the genetic variance necessary for kin recognition.

The origin and maintenance of polymorphic genetic recognition cues remains incompletely understood despite substantial theoretical and empirical research (e.g. [15,17,21-24]). Previous models of cue-dependent cooperation have treated the cooperative behaviour and the recognition cue phenotype either as products of a single locus (effectively a green beard locus; [15,25-29]) or separate loci [17,19,20,30]. In one such two-locus model, individual recognition alleles increased in frequency when in linkage disequilibrium with the cooperative allele, but were prevented from fixing by non-cooperating "freeloaders" with the same recognition allele [20]. Intermediate recombination rates produced cycles in which cooperation and multiple recognition alleles could coexist, suggesting that genetic recognition systems can remain somewhat stable under some conditions. By contrast, subsequent analytical models and simulations suggested that a very restrictive combination of high population structure, low recombination and frequent mutation is required to preserve recognition cue diversity [17]. Hence, the paradox of highly variable genetic recognition cues remains largely unresolved, at least under the assumption that recognition loci function only in the selection of social partners. The prevailing consensus is therefore that recognition loci likely have more than one function (i.e. they are pleiotropic), and that the pleiotropic function introduces negative frequency-dependent selection that preserves recognition cue diversity [9,15,17,23].

Such negative frequency-dependent selection could be provided by pathogens and parasites, for example when parasites evolve to preferentially infect hosts with common recognition cues (e.g. [17,31,32]). Some loci used in kin recognition also affect parasite resistance, notably the major histocompatibility complex (MHC) of vertebrates (e.g. [33]). Additionally, social insects use heritable chemical cues to identify colony members [12] as well as allospecific social parasites; these parasites may evolve to chemically mimic the commonest host genotypes [34-36]. Host-parasite interactions may similarly maintain cue diversity in parasitic species. *Copidosoma floridanum* parasitoid larvae identify and attack unrelated larvae present inside the same host using cues present on a membrane surrounding the larvae [37]. Common cues might therefore confer protection against unrelated larvae, but the membrane also defends larvae from the host’s immune system, which may select for rare phenotypes [38]. Rare cues have also been proposed to improve the precision of intra-specific recognition and thereby reduce the frequency of costly errors [9,14,29].

Recognition cues that inform social behaviour might also be used in the context of mate or gamete choice (e.g. [17,39]). A pleiotropic function in mate choice might preserve genetic polymorphism at recognition loci by at least four non-exclusive mechanisms.

Firstly, individuals with rare recognition cues might be attractive to or compatible with a greater proportion of potential mates, for example if individuals avoid inbreeding by discriminating against potential mates with similar recognition cues, or if matings between partners with the same recognition locus genotype are infertile [39]. Individuals with rare cues would therefore have a sexually-selected advantage that might counterbalance their disadvantage in social interactions. This hypothesis reflects the well-known population genetic result that disassortative mating can increase genetic diversity provided that it creates inequalities in mating success [40].

Secondly, disassortative mating with respect to recognition loci should increase their heterozygosity. Assuming that cooperation increases the fitness of common genotypes, this would increase the fitness of heterozygotes, promoting genetic polymorphism. This hypothesis [proposed in 15] assumes that cooperation occurs primarily or solely between individuals that share both alleles at the recognition locus (which we call "2-allele matching").

Thirdly, for species in which cooperation occurs between individuals sharing at least one recognition allele ("1-allele matching"), we suggest that the increase in heterozygosity caused by disassortative mating could provide "hiding places" for rare recognition alleles. Under 1-allele matching, rare alleles may receive substantial amounts of cooperation when sharing a body with a common allele. Disassortative mating causes rare alleles to exist as heterozygotes even more often than predicted under random mating. This could prevent rare alleles from being lost from the population, increasing the ability of mutation and migration to maintain recognition locus diversity [17].

Fourthly, we propose that disassortative mating for recognition cues may indirectly lead to disassortative mating for condition. This is because matings between individuals with common and rare cues should be more frequent under disassortative mating, and individuals with common cues will tend to be in better condition since they receive greater average payoffs in social interactions. Under disassortative mating, individuals with rare cues might tend to have a mating partner in better condition than themselves and *vice versa*, potentially improving the relative fitness of individuals with rare recognition alleles.

Previous models of genetic kin recognition have mostly assumed random mating and haploidy (precluding heterozygosity), so the effect of mating systems on Crozier’s paradox is currently unclear. Here, we explore in detail the hypothesis that mate choice affects the evolution of cooperation based on genetic recognition cues using individual-based simulation. We also review the available literature in order to evaluate the relative importance of mate choice in stabilising cue-dependent cooperation in diverse taxa.

## Methods

The individual-based simulation was inspired by simulations in Rousett and Roze [17]. In brief, we consider the evolution of cue-dependent cooperation in a finite, patch-structured population of sexually reproducing individuals that possess two loci that control recognition and cooperation respectively. In each generation, individuals potentially cooperate with their patch mates, then mate and produce offspring, some of which disperse to other patches. The key differences of our model are that mating within patches can be non-random with respect to the recognition locus, and individuals are diploid not haploid, facilitating investigation of the consequences of heterozygosity differences between mating systems.

Table 1 summarizes the parameters and their possible ranges. Patches contain *N* diploid hermaphrodites, the number of patches is given by the population size divided by *N*, and *N* is assumed to be even. The recognition locus has *k* possible co-dominant alleles, while the behaviour locus has a cooperative allele and a non-cooperative allele (where the degree of dominance can be specified; see below). Cooperation is contingent on the actor sharing either one or both recognition alleles with the recipient; we modelled such "1-allele matching" and "2-allele matching" systems separately. In each generation, all possible pairs of individuals within each patch meet, and if the actor is homozygous for the cooperative allele and is sufficiently similar to the recipient at the recognition locus, the condition of the recipient is increased by *b* at a net cost *c* to the condition of the actor. We therefore assume either that the recognition locus also causes individuals bearing the cooperative allele to only cooperate with others bearing similar recognition cues (in addition to producing the cue), or that genes causing such a preference are fixed at other loci (as in previous models; Jansen and van Baalen 2006; Rousset and Roze 2007). Heterozygous cooperators provide *h* times as much help as homozygous cooperators (where 0 ≤ *h* ≤ 1), such that they confer a benefit *bh* and pay a cost *ch*, while non-cooperator homozygotes never provide help.

**Table 1:** List of parameters in the model

**Parameter**	**Range**	**Description**
*m*	-1 to 1	Determines mating system. Disassortative: *m* < 0, random: *m* = 0, assortative: *m* > 0. Mating becomes increasingly random as *m* approaches 0
*b*	0 to ∞	Benefit of receiving cooperation
*c*	0 to ∞	Cost of performing cooperation
*x*	0 to ∞	Number of additional offspring produced when mating partners have different recognition locus genotypes
*N*	2 to Population size	Patch size; assumed to be even. Population size was set at 10,000 in all simulations
*k*	2 to ∞	Number of possible recognition alleles
*r*	0 to 0.5	Recombination rate between the recognition and behaviour loci
*d*	0 to 1	Probability that offspring disperse from the natal patch
μ	0 to 1	Mutation rate of both loci
*h*	0 to 1	Dominance of the cooperative allele; heterozygotes cooperate fully when *h* = 1, partially when 0 <*h* < 1, and never when *h* = 0

An individual’s net condition after the helping phase is equal to a base value, adjusted for the costs and benefits of cooperative acts performed and received. Base condition was set to *cN*, i.e. the cost of helping all individuals in the patch. Condition is therefore equal to *cN + b*_*sum*_*- c*_*sum*_, where *b*_*sum*_ and *c*_*sum*_ are the summed benefits and costs of cooperative actions received and performed by the focal individual; *c*_*sum*_ is zero for non-cooperators.

After the cooperation phase, individuals mate with a single partner from their patch, produce offspring and then die (i.e. we assume monogamy and non-overlapping generations). To explicitly model the effect of different mating systems on the persistence of cue-dependent cooperation, we allowed for non-random mating within patches. The mating system was determined by the parameter *m*, which ranges from -1 (strongly disassortative) to 1 (strongly assortative). When *m* = -1, individuals always mate with partners that share no recognition alleles whenever such partners are present in the same patch; once there are no such pairings left, individuals that have one allele in common are mated. The remaining unmated individuals are then mated at random. The reverse is true when *m* = 1, i.e. all individuals share one or both alleles with their mate whenever possible. For values between -1 and 1, there is a probability equal to 1–|*m*| that the "preferred" mating type will not occur; for example, for *m* = 0.25 we first pair up all the same-type individuals, and each pair mates with a probability 0.25. Those that do not mate then go on to be paired up with individuals with which they share one allele, and again mate with a probability of 0.25. All the remaining unmated individuals then mate with a random partner. Thus mating becomes increasingly random as *m* approaches zero, and is completely random when *m* = 0.

The number of offspring that are produced and survive until the migration phase is equal to the condition of one randomly selected parent (the "mother"), rounded down to the nearest integer. Mating pairs that are non-identical at the recognition locus produce an additional *x* offspring. Higher values of *x* disproportionately increase the fitness of rare recognition alleles, because rare genotypes are more likely to be involved in a dissimilar-type mating for any given *m*. We envisage *x* as representing the sum of the possible direct benefits (e.g. lower costs of mate searching or courtship) and indirect benefits (e.g. lower mortality of outcrossed offspring) of dissimilar-type matings. As *x* increases, the results of the model converge on those that would be obtained had we assumed that only disassortative matings are fertile. Disassortative mating is more likely to evolve if it is selectively advantageous, such that *x* and *m* will tend to be negatively correlated in nature.

Diploid offspring were produced assuming Mendelian inheritance. Recombination occurs between the two loci with probability *r* (0 ≤ *r* ≤ 0.5), and mutation with probability μ. Mutation converts a cooperative allele to a non-cooperative allele and *vice versa*, and converts a recognition allele to one of the other (*k* – 1) possible alleles. Each offspring has a probability *d* of dispersing to another randomly-selected patch; otherwise, it remains at the natal patch. After the migration phase, the populations in each patch are randomly culled down to the patch size *N*.

At the start of each simulation, all individuals had a random recognition locus genotype and were homozygous for the non-cooperative allele; the model therefore considers the origin of cooperation based on pre-existing, polymorphic recognition cues. Simulations were run for 50,000 generations with a population size of 10,000. Recognition locus diversity was measured using an index, calculated as $(1-\sum_{k=1}^{k}f_{k}{}^{2})/\left(1–\left(1/k\right)\right),$ where *f*_*k*_ is the frequency of the *k*^th^ allele. This index is zero when one allele has fixed and one if all *k* alleles are present at equal frequencies.

## Results

The primary findings of the model are: 1) disassortative mating and beneficial outbreeding can maintain cue-dependent cooperation over evolutionary time under certain conditions, and 2) mate choice interacts with other parameters in its effects on recognition locus diversity and the frequency of the cooperative allele.

**Figure 1:**
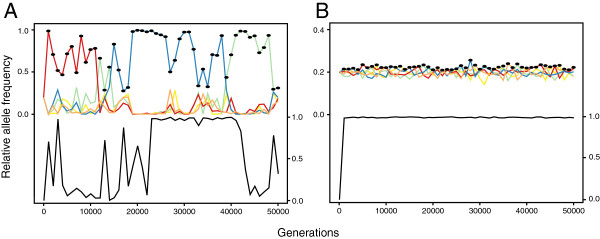
**Two illustrative runs of the individual-based simulation, showing allele frequencies at the recognition locus (coloured lines) and behaviour locus (black line).** Black dots mark the recognition allele with the highest frequency among individuals with at least one cooperative allele. Panel **A**: In this run, cooperation eliminated recognition locus diversity. The figure assumes the following parameter space: *x* = 6, *b* = 6, *c* = 1, *m* = 0, *N* = 6, *k* = 5, *r* = 0.05, *d* = 0.1, μ = 10^-5^, *h* = 1 and 2-allele matching, as in the centre of Figure 2B. Panel **B**: The cooperative allele invaded without depleting recognition locus diversity. The parameter space is the same as before except *b* = 3 and *m* = -1, as in the bottom left of Figure 2B.

Two illustrative runs of the model are shown in Figure 1. In Figure 1A (which assumes random mating), individual recognition alleles excluded nearly all of the others by hitchhiking to high frequency with the cooperation allele, before declining once non-cooperators bearing the same recognition allele became common. They were replaced by another dominant lineage of cooperators, which in turn succumbed to "freeloaders" and were replaced; this figure shows Crozier’s paradox in action. By contrast, in Figure 1B (which assumes disassortative mating), multiple lineages of cooperators persisted indefinitely at largely equal frequencies.

**Figure 2:**
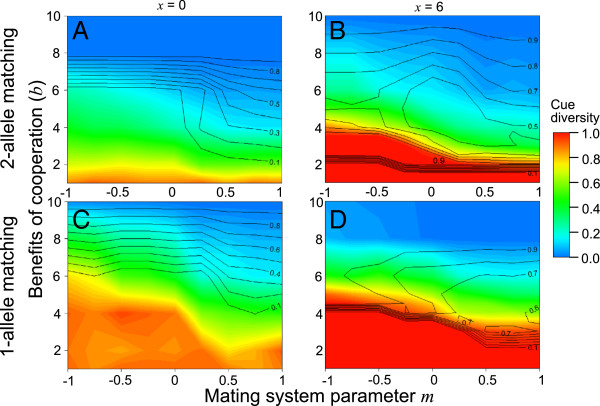
**Recognition locus diversity and cooperation are affected by the benefit of cooperation to the recipient, the mating system and the mode of recognition.***m* < 0 corresponds to disassortative mating, while *m* > 0 represents assortative mating. Panels **A** and **C**: if outcrossed matings do not affect the number of surviving offspring (*x* = 0), cooperative behaviour typically erodes recognition locus diversity. Panels **B** and **D**: if outcrossed matings produce more offspring (*x* > 0), both cooperation and variable recognition cues can persist. All figures assume the following parameter space: *N* = 6, *c* = 1, *k* = 5, *r* = 0.05, *d* = 0.1, μ = 10^-5^ and *h* = 1.

**Figure 3:**
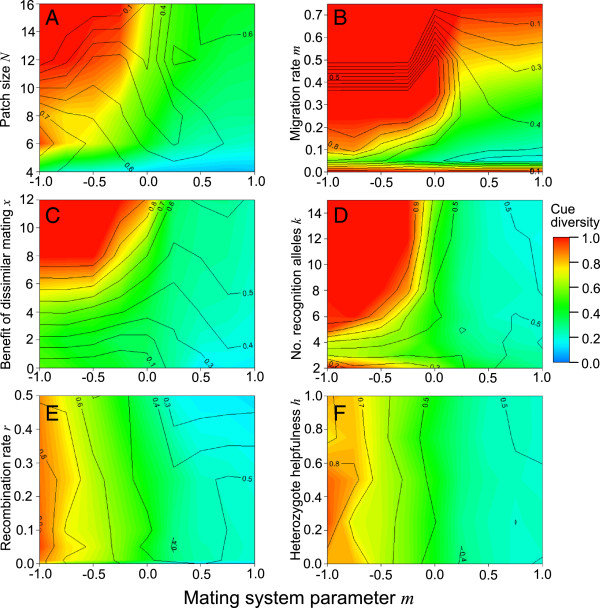
**The effects of mating system on the evolution of cue-dependent cooperation interacted with those of other model parameters.** Patch size (*N*), dispersal rate (*d*), the benefit of disassortative mating (*x*) and the number of recognition alleles (*k*) all interact with mating system (Figures 3**A**-3**D**), but recombination rate (*r*) and the behaviour of individuals heterozygous for the cooperative allele (*h*) do not (Figures 3**E** and 3**F**). Unless stated otherwise, all figures assume the following parameter space: *x* = 6, *b* = 4, *c* = 1, *N* = 6, *k* = 5, *r* = 0.05, *d* = 0.1, μ = 10^-5^, *h* = 1 and 2-allele matching.

Figures 2 and 3 examine the effects of the model’s parameters on recognition locus diversity and the frequency of cooperation at equilibrium. Redder colours denote higher recognition locus diversity, while the frequency of the cooperative allele is shown by contour lines; parameter spaces favouring the evolution and maintenance of cue-dependent cooperation thus have red colours and high values on the contour lines. The figures show the average values of the last 1000 generations (n = 10 replicates per parameter space; 45–90 parameter combinations per figure). The high similarity of neighbouring parameter spaces indicates that the simulations produced consistent results, and thus that 10 replicates per parameter space was sufficient. Moreover, the standard error of the output of the 10 runs was low: 0.019 for recognition locus diversity and 0.023 for the frequency of the cooperative allele (averaged across all parameter spaces examined).

### Maintenance of cue-dependent cooperation by mate choice

We first held all parameters constant except for *b*, *m* and *x*, to test how the mating system affects the evolution of cue-dependent cooperation. Assuming 2-allele matching and no direct fitness consequences to mate choice (*x* = 0; Figure 2A), recognition locus diversity was lost whenever cooperation invaded, even under disassortative mating. With 2-allele matching and *x* > 0 (i.e. dissimilar-type matings produce more surviving offspring; Figure 2B), negative frequency-dependent selection arose at the recognition locus because individuals with rare recognition alleles produce an additional *x* offspring more often, which in some cases maintained maximal values of cue diversity and cooperation. Disassortative mating increased the parameter space under which cue-dependent cooperation was able to persist. Note also that when the *b*:*c* ratio was low, cooperation did not evolve (as expected from inclusive fitness theory [1]) but recognition locus diversity persisted at maximal values. This shows that recognition cue diversity remained at high levels under mutation-drift balance when selection from cooperation was absent under our assumptions, such that all non-red parts of the figures signify the erosion of diversity by cooperation (Crozier’s paradox).

Figures 2C and 2D show the same parameter space as Figures 2A and 2B, but assume 1-allele matching. The cooperative allele required a higher *b:c* ratio to invade and persist with this less stringent mode of recognition, which causes cooperators to help non-cooperators more frequently. Comparing Figures 2A and 2C, one can see that 1-allele matching coupled with disassortative mating creates a parameter space under which fairly high (green) values of cue diversity and cooperation can persist even when *x* = 0, which is not true of 2-allele matching. We therefore found evidence that disassortative mating paired with 1-allele matching can counteract Crozier’s paradox even if inbred and outbred matings are equally fit. This effect occurs because disassortative mating increases the heterozygosity of the recognition locus, and 1-allele matching means that heterozygotes containing one rare and one common recognition allele will experience at least as much cooperation as individuals homozygous for the common allele. Selection against rare alleles therefore becomes weaker, promoting polymorphism at mutation-selection balance. However, we found little support for Crozier’s [15] prediction that disassortative mating can preserve polymorphic recognition cues by increasing the frequency (and therefore fitness) of heterozygotes. If this effect were strong, one might expect diversity to sometimes be preserved under 2-allele matching, disassortative mating and *x* = 0, yet it was always lost once cooperation invaded (Figure 2A).

Disassortative mating also caused the cooperative allele to be maintained at lower frequencies than under random or assortative mating (Figure 2). This result possibly arose because disassortative mating leads to relatively more matings between individuals with common and rare recognition genotypes, which tend to be high-condition and low-condition respectively. Because the cooperative allele is typically in linkage disequilibrium with common recognition alleles (Figure 1; see also [17,20]), this negative correlation between parental qualities simultaneously lessens the fitness gap between common and rare recognition alleles, and between the cooperative and non-cooperative alleles. Conversely, assortative mating produces a positive correlation between parental qualities, which benefits the most common recognition alleles, as well as the cooperative allele (which is typically in linkage disequilibrium with common recognition alleles).

### Interactions between mate choice and other parameters

Because mate choice was found to have less effect on the maintenance of cue-dependent cooperation when *x* = 0, we set *x* = 6 for subsequent simulations (except those in Figure 3C), i.e. we assumed that matings between individuals with non-identical recognition genotypes produce additional offspring, equivalent to being helped 6/*b* times. We also assumed 2-allele matching.

Population viscosity generally fostered the evolution of cue-dependent cooperation. Smaller patches and intermediate dispersal rates produced the highest number of cooperators, and both patch size and dispersal rate interacted with mating system in their effects on the evolution of cue-dependent cooperation (Figures 3A and 3B). Cue-dependent cooperation did not persist at any patch size under assortative mating under the present assumptions, but diverse cues and relatively high rates of cooperation were maintained if mating was disassortative and patches were small. Dispersal rate had a stronger effect on the frequency of cooperation under disassortative mating than assortative mating; disassortative mating allowed cue-dependent cooperation to persist even in relatively unstructured populations. As expected, cooperation did not persist when dispersal rates were close to zero, because competition among kin counteracts the inclusive fitness benefits of cooperation [41,42].

The amount of extra offspring produced by mating pairs with dissimilar recognition alleles (*x*) was positively related to both recognition locus diversity and the frequency of cooperation, especially under disassortative mating (Figure 3C). This result means that if rare recognition alleles are advantageous in sexual interactions, cue-dependent cooperation is more likely to persist. The interaction with mating system arises because the benefit of dissimilar matings is obtained more often when individuals tend to mate disassortatively. The precise value of *x* needed to stabilise cue-dependent cooperation is presumably a function of the *b:c* ratio, mating system and current frequency of cooperation.

The number of recognition alleles present (Figure 3D) was positively related to both diversity and the frequency of cooperation, up to an asymptote (as in [17]); this effect was especially pronounced under disassortative mating. The cooperative allele also failed to invade when the number of possible recognition alleles was low (*k* = 2), because the recognition locus provided inadequate information on relatedness at the cooperation locus for cooperation to be selectively favoured in this parameter space.

Strong or complete linkage between the two loci caused a drop in recognition locus diversity (Figure 3E, region: 0 <*r* < 0.01); however, varying the recombination rate from 0.01 to 0.5 had little effect on equilibrium allele frequencies. With very low recombination, recognition alleles can remain associated with the cooperative allele for longer periods, and can therefore displace more of the other recognition alleles before linkage disequilibrium between the common recognition allele and the cooperative allele decays via recombination and mutation (replicating previous models [17,20]). There was no evidence that recombination rate and mating system interacted in their effects on cue-dependent cooperation.

We also investigated the effects of dominance at the behaviour locus, but found that the phenotype of heterozygotes (*h*) had no effect on the equilibrium recognition locus diversity or the frequency of cooperation (Figure 3F).

## Discussion

The simulations reaffirm that genetic kin recognition presents an evolutionary paradox [15], and demonstrate that disassortative mating provides a potential resolution.

Disassortative mating was especially powerful at maintaining cue diversity when matings between individuals with different recognition cues were assumed to be more fecund (*x* > 0). Individuals with rare cues reaped this "bonus" fecundity more often, particularly under disassortative mating. Even when all mating types were equally fecund (*x* = 0), meaning that individuals with rare cues received no direct fitness benefits, disassortative mating helped to preserve diversity by increasing the frequency of recognition locus heterozygotes, which increases heterozygote fitness. This effect is strongest with 1-allele matching, which turns heterozygotes into refugia for rare alleles.

There was also evidence that disassortative mating preserves rare alleles by increasing the frequency of matings between high quality (common-cue) and low quality (rare-cue) individuals. The four mechanisms discussed in the introduction together rescued cue-dependent cooperation under a range of parameter spaces. Their relative importance likely varies under different assumptions, but overall we found strong support for the hypothesis that disassortative mating can resolve Crozier’s paradox.

As in previous theoretical treatments [17,20], we found that population structure was a key determinant of the invasion and persistence of cue-dependent cooperation. We further showed that the effects of mate choice interacted with those of population structure, affecting the range of parameters under which cue-dependent cooperation could evolve and persist. For example, cue-dependent cooperation remained stable under most dispersal rates with disassortative mating, a narrow range of dispersal rates with random mating, and was never stable under assortative mating. Similarly, the evolution of cue-dependent cooperation is constrained if only a small number of distinct recognition alleles can exist [17], but disassortative mating allows cooperation to invade for smaller numbers of alleles.

### Empirical evidence that disassortative mating increases diversity at recognition loci

The hypothesis that disassortative mating stabilises cue-dependent cooperation has three principal assumptions: 1) the recognition cues that facilitate assortative cooperation also affect mate choice, 2) individuals mate disassortatively with respect to their recognition cues, and 3) recognition cues are heritable and are under positive frequency-dependent selection from social behaviours, such that cue variation could in principle be eroded as predicted [15].

Evidence that kin recognition cues are simultaneously used in sexual and social interactions is fairly abundant; certainly, inbreeding avoidance and positive effects of outbreeding are very common [43]. The heritability of recognition cues has also been documented a number of times, though the relative importance of genetic and environmental variation likely differs among taxa.

We will now review the empirical evidence that polymorphism at recognition loci can be maintained by selection on a pleiotropic function in mate choice, with the aims of evaluating the relative importance of this mechanism across taxa and identifying gaps in our knowledge. These data are summarised in Table 2; much of the data may seem inconclusive, but it is out hope that this review will highlight gaps in our knowledge and stimulate further work.

**Table 2:** Review of empirical evidence that disassortative mating contributes to the maintenance of genetic variation in kin recognition cues

**Taxon**	**Cue used to facilitate assortative cooperation**	**Inbreeding avoidance/disassortative mating?**	**Cue used in mating choice**	**Potential for resolution of Crozier’s paradox by disassortative mating**	**References**
Slime mould *Dictyostelium discoideum*	TgrB1 and TgrC1 surface proteins	Yes	*mat* locus	No	Benabentos et al. 2009 [13], Bloomfield et al. 2010 [49], Hirose et al. 2011 [46]
Fungi *Neurospora crassa, Aspergillus heterothallicus, Sordaria brevicolus*	*MAT* loci	Yes	*MAT* loci (lipopeptide pheromones)	Yes	Shiu and Glass 1999 [51], Aanen et al. 2008 [9], Hall et al. 2010 [52]
Fungi, other species	Other heterokaryon incompatibility loci	Yes	*MAT* loci (lipopeptide pheromones)	Possibly	Shiu and Glass 1999 [51], Aanen et al. 2008 [9], Hall et al. 2010 [52]
Colonial ascidians *Botryllus schlosseri* and *Hydractinia symbiolongicarpus*	Histocompatibility locus A	No	n/a	No	Grosberg and Quinn 1986, Grosberg and Hart 2000 [39], Rosengarten and Nicotra 2011 [14]
German cockroach *Blattella germanica*	CHCs	Yes	CHCs	Yes	Lihoreau et al. 2007 [59]; 2008 [60]; Lihoreau and Rivault 2008 [61]; 2010 [62]
Halictid bee *Lasioglossum zephyrum*	Lactones and/or CHCs	Yes	Lactones and/or CHCs	Possibly	Greenberg 1979 [72], Smith 1983 [68], Smith and Wenzel 1988 [73]
Social wasps *Polistes dominulus* and *P*. *fuscatus*	CHCs	Yes	Unknown	Possibly	Ryan and Gamboa 1986 [76], Gamboa 2004 [74], Liebert et al. 2010 [77]
Social wasp *Ropalidia marginata*	CHCs	No	n/a	No	Shilpa et al. 2010 [78]
Bumble bees *Bombus spp.*	Probably CHCs	In some species	Unknown	Possibly	Foster 1992 [69], Whitehorn et al. 2009 [79], Martin et al. 2010 [75]
Ants *Leptothorax gredleri* and *Linepithema humile*	CHCs	Yes	Probably CHCs	Possibly	Keller and Passera 1993 [70], Oppelt et al. 2008 [71], van Zweden and d’Ettorre 2010 [12]
Tuatara *Sphenodon punctatus*	MHC	Yes	MHC	Weak	Miller et al. 2009 [99]
Zebrafish *Danio rerio*	Odour cues	Yes	MHC-derived odours	Possibly	Gerlach and Lysiak 2006 [88], Gerlach et al. 2008 [89]
Arctic charr *Salvelinus alpinus*	MHC-derived odours	Yes	MHC-derived odours	Yes	Olsén et al. 1998 [95], Skarstein et al. 2005 [98]
Atlantic salmon *Salmo salar*	MHC-derived odours	Yes	MHC-derived odours	Yes	Landry et al. 2001 [90], Rajakaruna et al. 2006 [96]
Long-tailed tit *Aegithalos caudatus*	Contact calls	Yes	Unknown	Possibly	Hatchwell et al. 2000 [111], Sharp et al. 2005 [109]
Mouse *Mus musculus*	MHC-derived odours	Yes	MHC-derived odours	Yes	Yamazaki et al. 1976 [84], 1988 [85], 2000 [83], Potts et al. 1991 [87], Manning et al. 1992 [82]
Naked mole rat *Heterocephalus glaber*	Odour cues	Yes	Odour cues	Possibly	Clarke and Faulkes 1999 [114]
Mandrill *Mandrillus sphin*x	Odour cues	Yes	MHC-derived odours	Possibly	Charpentier et al. 2007 [11], Setchell et al. 2010 [116], 2011 [117]
Human *Homo sapiens*	Facial cues	Yes	Facial cues	Yes	DeBruine 2005 [122], Bailenson et al. 2008, DeBruine et al. 2008 [119], Krupp et al. 2008 [119], Nojo et al. 2011 [123]

We list cases in which a cue used to facilitate assortative cooperation has been identified, state whether inbreeding avoidance or disassortative mating has been reported and examine whether the cues used in social and mate choice contexts are the same, potentially resolving Crozier’s paradox. CHCs: cuticular hydrocarbons, MHC: major histocompatibility complex.

#### *Microorganisms and marine invertebrates*

In the social amoeba Dictyostelium discoideum, individuals aggregate to form a multi-cellular "slug" when starved. This behaviour is cooperative because many of the cells die to produce a stalk, which lifts the asexual spores formed by the remaining cells to aid their dispersal [44]. Cells are more likely to form a slug with related cells [45], and kin recognition is mediated by at least two transmembrane proteins encoded by the highly polymorphic genes tgrB1 and tgrC1 [13,46]. D. discoideum also reproduces sexually; mating is disassortative with respect to a mating type locus, which determines whether two strains can mate, and sexual recombination appears frequent in natural populations [47]. Early reports suggested that the mating type locus was either the same as or linked to the loci controlling slug formation [48], implying that negative frequency dependent selection on the mating type locus could maintain cue polymorphism. However this conclusion was later disproved: tgrB1 and tgrC1 do not directly affect mating type [49] and they are on a different chromosome to the mating type locus [50]. Disassortative mating is therefore unlikely to be important in the maintenance of allelic diversity at tgrB1 and tgrC1.

Fungi possess genetic recognition "heterokaryon" loci that allow somatic fusion between sufficiently similar genotypes [9]. Assortative somatic fusion is a ubiquitous form of cooperation amongst fungi, and diversity at these loci is therefore predicted to be unstable. Several pleiotropic functions of these loci have been proposed to maintain diversity including selection from sexual reproduction [9], as the mating type locus also functions as an allorecognition locus for somatic fusion in a number of species [51]. Sequence data for these loci suggest that they are under balancing selection in several species in the Neurospora/Sordaria complex, consistent with maintenance of polymorphism by selection on the mating functions of the loci [52].

The disassortative mating hypothesis was explicitly tested in the colonial ascidian Botryllus schlosseri [39]. This organism has a single recognition locus with >100 alleles; neighbouring individuals sharing one or both alleles undergo somatic fusion, which is thought to be beneficial among clonemates [14]. Gametes from individuals of known genotype were mixed to investigate if dissimilar-type crosses produced more offspring [39]. All crosses yielded the same amount of offspring, suggesting that the recognition locus does not affect "mate choice" (i.e. egg-sperm recognition), and therefore that recognition locus diversity is maintained by other factors in this taxon. The mechanisms promoting diversity are uncertain, although recognition locus homozygotes have high juvenile mortality, suggesting that the kin recognition locus has a pleiotropic effect [53]. Our model also predicts that the 1-allele matching recognition should slow the cue-eroding effects of selection from fusion. Additionally, fusing with non-clonemates can be harmful, because individuals may have their germline replaced by that of the partner [14]. Fusion might therefore impose negative frequency-dependent selection on the recognition locus, because rare types will fuse with non-clonemates less often. The net direction of selection from fusion should depend on the net inclusive fitness effects and relative frequencies of fusion between clone mates and non-clonemates.

#### *Insects*

In many species of insects, kin recognition is mediated by cuticular hydrocarbons (CHCs), a waxy layer present on the body surface [12]. Hydrocarbon production has a strong genetic component in diverse insects (e.g. [54-58]). Some of the strongest evidence that CHCs have a dual role in kin recognition and mate choice comes from the group-living cockroach Blattella germanica. Behavioural experiments that disentangle genetic similarity and familiarity have found that individuals prefer to aggregate with kin in the absence of sex pheromone (a putatively cooperative behaviour), but that both sexes apparently avoid inbreeding using olfactory genetic kin recognition [59-62].

In ants, CHCs are the main cue used to identify and aggressively reject non-nestmates [12], which tend to be non-relatives. Because ants identify non-nestmates by detecting novel odours [63,64], rare CHC phenotypes may receive more aggression from neighbouring colonies, leading to erosion of genetic variation for CHC production. In support of this prediction, inter-colonial aggression in the Argentine ant Linepithema humile is polarised, such that genetically diverse colonies are aggressed by colonies with lower diversity but are comparatively non-aggressive in return [18]. A loss of genetic polymorphism at recognition loci may explain the origin of "supercoloniality" in ants, in which whole populations are mutually cooperative [65]. In our model (Figure 1A) and others [20], losses of recognition locus diversity were often followed by a decline in cooperation, but ants are likely prevented from losing cooperative traits by strong evolutionary constraints [65]. Many hymenopterans possess complementary sex determination and should therefore be selected to mate disassortatively (see [66]), and inbreeding can be costly despite the purging effects of haplodiploidy [67]. A number of behavioural studies have found evidence for inbreeding avoidance in social Hymenoptera (e.g. [68-71]), but the role of CHCs in mate choice has yet to be experimentally verified. However, Oppelt et al. (2008) found that Leptothorax gredleri ant queens prefer to mate with non-nestmate males and that the CHCs of both sexes are colony-specific, suggesting that CHCs encode sufficient information for mate choice.

In the primitively eusocial bee Lasioglossum zephyrum, related females are more likely to be accepted as a social partner regardless of familiarity, suggesting heritable recognition odours [72]. Moreover, males were less attracted to females that were close relatives of previously encountered females, suggesting mate choice based on learned genetic cues [68]. Macrocyclic lactones from females’ Dufour’s glands correlate with relatedness and may be used in both selection of social partners and mates [73], possibly in combination with CHCs. Wasps and bumble bees also apparently use CHCs to distinguish kin from non-kin [74,75], though the role of CHCs in mate choice is quite poorly understood. Polistes fuscatus paper wasps mate preferentially with non-nestmates, which are apparently identified by learned genetic recognition cues [76], and P. dominulus males displayed a preference for non-nestmate females [77]. However, no such mating preference was detected in Ropalidia marginata [78]. Bombus frigidus and B. bifarius bumble bees showed a mating preference for non-nestmates, but B. californicus and B. rufocinctus mated indiscriminately [69]. Additionally, B. terrestris reproductives took longer to mate with siblings than with non-relatives [79].

One assumption of Crozier’s paradox, that social behaviours cause positive frequency-dependent selection at recognition loci, has been questioned in social insects. Ratnieks (1991) argued that because nestmate recognition cues are used to identify and exclude conspecific intruders, colonies with rare recognition alleles might actually have higher fitness because they are better at identifying enemies. The relative frequency of robbing versus other forms of social interactions differs between taxa, and it is presently unclear whether selection acts to increase or decrease recognition cue diversity in most species (but see [18]).

#### *Vertebrates*

The MHC loci of vertebrates play a critical role in the immune system but also function in mate choice, such that MHC diversity may be generated by both parasite pressure and disassortative mating (reviewed in [80,81]). Common MHC alleles may be more susceptible to parasites, which evolve to preferentially infect the most commonly encountered host genotypes, providing a rare-allele advantage [32]. Disassortative mating with respect to MHC genotype has been reported in diverse taxa, and may offer fitness advantages such as increased/optimal MHC diversity or increased genome-wide offspring heterozygosity [80,81].

The role of MHC-based recognition in social and cooperative behaviour is understudied relative to mate choice. Putative examples have been observed in mice Mus musculus; females are more likely to nest with, and thus nurture the offspring of, individuals of a similar MHC genotype [82]. Also, adults preferentially retrieve pups with a similar MHC genotype to themselves when presented with scattered pups [83]. Strong evidence for disassortative mating based on MHC is provided by studies of inbred mouse lines differing solely in their MHC genotypes [84-86]. Mates from the other line were preferred, but this preference was reversed in mice that had been reared by foster mothers from the other line. Young mice therefore imprint on the MHC-based odours of family members and later develop a sexual aversion to familiar odours, and preferences for MHC-derived odours are opposite in mating and social contexts. Disassortative mating based on MHC genotype has also been demonstrated in semi-natural populations [87].

In the zebrafish Danio rerio, juveniles prefer the odour of unfamiliar kin over unfamiliar non-kin when shoaling, but females reverse this tendency when sexually mature [88]. The odour cues involved are unknown, although they are learned from siblings early in life, and MHC-derived odours are a likely candidate [89]. MHC-based mate choice is well-documented in other fish [90-93], and preferential shoaling with familiar individuals [94] or MHC-similar individuals [95-97] has been found in Gasterosteus aculeatus, Salvelinus alpinus, Salmo salar and S. trutta. In S. alpinus, MHC-heterozygous males were more successful in sperm competition than homozygotes, and the data imply that eggs may select sperm based on the male’s MHC genotype [98]. S. salar also mates disassortatively for MHC genotype [90].

Amongst reptiles, tuataras (Sphenodon punctatus) apparently use the MHC to avoid kin both in mate choice and in territorial fights [99], although the effect was relatively weak. Rare phenotypes should in theory thus receive more mating success and more territorial challenges as compared to common phenotypes.

Kin recognition based on odour has been reported in birds [100,101] and may affect their mate choice [102-104]. Although there is no direct evidence that genetic kin recognition is used to inform cooperative behaviour, males have been found to preferentially lek next to relatives in a few species [105-108], in at least one case independently of familiarity [106]. Joint lekking is thought to increase mating success and may therefore be viewed as a form of cooperation with the potential to deplete recognition cue diversity. The recognition systems underlying kin-biased joint lekking remain to be found.

Auditory recognition cues may also be used to inform cooperative decision-making in birds. In the cooperatively breeding long-tailed tit (Aegithalos caudatus), individuals distinguish kin from non-kin by their calls, and preferentially become helpers at the nests of relatives [109]. Vocalizations are learned in the juvenile stage from the provisioning parents, and are in that sense heritable; moreover, the identity-signalling song component of zebra finches has a genetic component [110]. There is evidence of inbreeding avoidance in long-tailed tits [111], but the role of song in this context is unconfirmed. Kin-biased helping has been reported in a number of other cooperatively breeding vertebrates (e.g. [112]), but the cues involved are usually unknown.

Naked mole rats (Heterocephalus glaber) form eusocial colonies containing a single reproductive female and many sterile helpers [113]. Cooperation is therefore directed solely towards kin, but when selecting mates, females choose individuals bearing unfamiliar odours [114]. Mate choice appears to involve sexual imprinting, but the nature and heritability of the odours used for discrimination remain to be elucidated.

There are also reports of disassortative mating for MHC genotype in primates [115,116]. For example, Mandrillus sphinx mates disassortatively with respect to MHC genotype (which correlates with olfactory cues), and MHC diversity predicts reproductive success [116,117]. Assessment of relatedness by olfactory cues may also be used in the selection of social partners in this species [11]. In humans, evidence for MHC-based mate choice is mixed, although the majority of studies imply a preference for MHC-dissimilar partners [118]. However, we know of no strong evidence that MHC-derived odours affect cooperative decision-making or the choice of social partners. By contrast, humans are able to infer relatedness from facial cues, and the degree of facial similarity between the actor and recipient has been experimentally linked to a range of cooperative behaviours [119-121]. Moreover, men and women rate opposite-sex faces digitally manipulated to resemble themselves or their close relatives as less attractive, suggesting disassortative mating for facial cues [120,122,123].

## Conclusions

Our model and the available empirical data suggest that selection from disassortative mating may be an important mechanism maintaining variation in genetic recognition cues across diverse taxa. No recognition loci have been definitively shown to facilitate both assortative cooperation and disassortative mating, although there are many examples in which the data are consistent with this interpretation. We hope that future research will be able to tackle the difficult next step: explicit quantification of the relative strength and direction of selection acting on recognition loci from different sources, namely social interactions and pleiotropic functions such as mate choice.

## Competing interests

The authors declare they have no competing interests.

## Author contributions

LH performed the simulations and wrote the manuscript, JSvZ gathered data for the literature review and co-wrote the manuscript, TAL and PdE co-wrote the manuscript. All authors read and approved the final manuscript.
